# Meeting report of the 2nd Lugano ExoDay: extracellular vesicles as next-generation clinical biomarkers and therapeutic agents

**DOI:** 10.20517/evcna.2022.17

**Published:** 2022-07-29

**Authors:** Carolina Balbi, Marina Cretich, Lucio Barile

**Affiliations:** ^1^Istituto Cardiocentro Ticino, Laboratories for Translational Research, Ente Ospedaliero Cantonale, Bellinzona 6500, Switzerland.; ^2^Istituto di Scienze e Tecnologie Chimiche “Giulio Natta” (SCITEC) - Consiglio Nazionale delle Ricerche, Milano 20100, Italy.; ^3^Faculty of Biomedical Sciences, Università della Svizzera italiana, Lugano 6900, Switzerland.

## INTRODUCTION

The *2nd Lugano ExoDay* was the second edition of a scientific initiative in southern Switzerland with a focus on innovations, guidelines, and pitfalls of extracellular vesicle (EV) research. This periodical meeting in Lugano intends to actively involve and bring into focus EV experts and scientists at the early career stage, driving interactions and promoting collaborations. The symposium was supported by the Swiss National Foundation (SNF) under the funding scheme of “Scientific Exchanges”, which is designed for researchers who want to host a highly relevant scientific event in Switzerland and invite experts from abroad. For the second year, the SNF grant was awarded to Istituto Cardiocentro Ticino-EOC Ente Ospedaliero Cantonale, who led the organization of the symposium.

The *2nd Lugano ExoDay* was a joint event with the first workshop of a pan-European consortium carrying out the MARVEL EU-funded project. The symposium benefited the consortium by disseminating the project’s concept, meeting with potential end-users or companies, and networking for future collaborations.

All participants had an in-depth overview of technical requirements and advances in isolation, characterization, and potential clinical application of EVs. Three keynote lectures of invited internationally renowned EV scientists were combined with eleven presentations of young scientists selected from the abstracts, plus a dedicated MARVEL talk giving an overview of the project. The program was framed by a poster session (displaying twelve posters), including an industrial exhibition. The best oral and poster presentations were awarded with prizes sponsored by *Extracellular Vesicles and Circulating Nucleic Acids* (*EVCNA)*.

The *2nd Lugano ExoDay* was one of the first in-person meetings in Switzerland since the start of the pandemic outbreak. All attendees showed a natural disposition toward interpersonal interaction, thus providing an enjoyable atmosphere. Thanks to all participants, the organizing team, and the supporting staff, the meeting offered a context for productive discussions. We report on the experts’ lectures and summarize the main scientific content in the following. Abstracts of oral and poster presentations are published in the present Special Issue.

### Keynote lectures

**Edit Buzàs** (Department of Genetics, Cell- and Immunobiology, Semmelweis University Budapest, Hungary) held the first keynote lecture. Prof. Buzàs, the newly nominated President of the International Society of Extracellular Vesicles (ISEV), performed an exciting lecture on the basic biology and features of EVs. She elucidated the different mechanisms of release for different classes of vesicles and pointed out how difficult it is to define EVs’ biogenesis. She then presented the new fascinating theory about the EV protein corona formation, recently published by her research group^[1]^. In particular, she showed how EVs from THP1 cells (a human leukemia monocytic cell line) with an external plasma protein cargo induced an increased expression of TNF‐α, IL‐6, CD83, CD86, and HLA‐DR of human monocyte‐derived dendritic cells, while EV‐free protein aggregates and "clean-EV" has no effect^[2]^. Prof. Buzàs’s lecture was an excellent and well-presented overview of the complexity of extracellular vesicles.

**Michele De Palma** (EPFL Swiss Federal Institute of Technology in Lausanne, School of Life Science, Lausanne, Switzerland) presented the second lecture. The speaker addressed basic questions regarding the role of tumor-released EVs in mediating the pro-metastatic effects of chemotherapy. He showed advanced and elegant research in his laboratory, providing evidence that two classes of cytotoxic drugs are broadly employed in preoperative (neoadjuvant) breast cancer therapy, elicit tumor-derived EVs with enhanced pro-metastatic capacity. Mechanistically he showed that chemotherapy-elicited EVs are enriched in annexin A6 (ANXA6), a Ca^2+^-dependent protein that promotes NF-κB-dependent endothelial cell activation, Ccl2 induction, and Ly6C+CCR2+ monocyte expansion in the pulmonary pre-metastatic niche to facilitate the establishment of lung metastasis^[3]^. Genetic inactivation of Anxa6 in cancer cells or Ccr2 in host cells blunts the pro-metastatic effects of chemotherapy-elicited EVs. ANXA6 is detected and potentially enriched, in the circulating EVs of breast cancer patients undergoing neoadjuvant chemotherapy.

**Benedetta Bussolati **(Department of Molecular Biotechnology and Health Sciences, University of Turin, Italy) held the third lecture. Prof Bussolati, the current President of the Italian Society of Extracellular Vesicles (EVIta), talked about one of the most exciting aspects of EV research: the therapeutic application of EVs. She well explained the evidence of the role of EVs derived from mesenchymal stem cells as a therapeutic agent, in particular showing how the scientific community shifted from cell therapy to the paracrine therapy paradigm. She then presented different supporting data, in particular on the beneficial role of EVs in the acute kidney disease (AKI) model^[4]^.

### MARVEL overview

MARVEL project (www.marvel-fet.EU), grant number 951768, started on 1 November 2020 and will end by 28 February 2023. It is coordinated by CNR (the National Research Council of Italy) and has the participation of six partners: two units, Università Vita-Salute San Raffaele (Italy) and Istituto Cardiocentro Ticino; Ente Ospedaliero Cantonale (Switzerland); and three SMEs, HansaBiomed from Estonia, PaperDropDX from Spain, and AMIRES from the Czech Republic. The main focus of MARVEL is to introduce a paradigm shift in affinity isolation of EVs from the use of antibodies to the use of peptides. Being well-established and totally synthetic molecules, peptides can overcome the major limitations inherent to using antibodies in affinity isolation, exceeding the analytical scale, such as high costs, batch-to-batch variation, and limited versatility of chemical manipulation. In particular, MARVEL is introducing membrane-sensing peptides (MSP)^[5]^ as novel ligands for the size-selective capturing of small EV. These ligands are “universal” because the capture of EVs is unbiased by differential protein expression and only exploits the general features of EV membranes such as high curvature, electrostatic charge, and lipid packing defects. In parallel, MARVEL is developing specific peptide probes (SPP) for EV-associated biomarkers to enrich clinically relevant EV subpopulations selectively. The versatility and modularity in scaling-up of these peptide-based systems are being demonstrated on medium/large sample volumes in two appropriate settings: (1) the manufacturing of GMP-compliant EVs as a medicinal product for cardiac repair with the collaboration of Istituto Cardiocentro Ticino; and (2) laboratory-scale urine-based liquid biopsy for bladder cancer stratification and monitoring in partnership with Università Vita-Salute San Raffaele.

MARVEL aims to impact the field of EVs by empowering the sustainability of EV use in both regenerative medicine and diagnostics. Such empowerment is expected to increment the readiness level of EV technologies and endow them with clinical-grade maturity. In line with the spirit of the “Transition” program funded by the EIC (European Innovation Council), MARVEL targets a technology readiness level (TRL) equal to 6 and envisages commercial exploitation of its results.

During the meeting, the project consortium, the concept, and the general aims were introduced by the coordinator Marina Cretich (CNR), whereas the Workpackage leader Alessandro Gori (CNR) provided details on the design and development of the peptide probes developed within MARVEL. In particular, an overview of MSP’s model of action and ways to generate a second generation of membrane binding probes was presented with emphasis on the scalability and versatility of peptide technology, which can be integrated into different EV isolation platforms including micro-analytical devices (biosensing chips and lateral flow test strips) and isolation tools such as beads, chromatography resins, and tangential flow filtration membranes.

### Main scientific content 

The scientific program included oral presentations that focused on potential theranostic (therapeutic and diagnostic) applications of EVs, interspersed with technical reports on innovative approaches for isolation and discrimination of different vesicle subpopulations. 

Talks focusing on the therapeutic application included mainly research performed using EVs isolated from conditioned medium of mesenchymal primary human cell lines: EVs from adipose tissue (ADSC) and bone marrow (BMSC) presented similar characteristics in terms of size, concentration, and marker expression, but they exhibited different characteristics in terms of protein content and functional effects. ADSC-EVs over-expressed pro-angiogenic factors in comparison to the BMSC counterpart (Cansu Gorgun, University of Genova, Italy). MSC from the connective tissue of human umbilical cords, the so-called Wharton’s jelly, are enriched in small non-coding RNA (miRNAs) that are able to target TP53 transcript and induce therapeutic benefit in preclinical models of premature birth-related white matter injury (Vera Tscherrig, University of Bern, Switzerland). The talk by Vera Tscherrig was selected as “Best Oral Presentation”.

Cardiac-specific mesenchymal cells cultured using a Good Manufacturing Practice (GMP)-compliant method in xeno-free conditions secrete EVs that are capable of preserving heart function in rat and pig models of acute myocardial infarction (Elena Provasi, Istituto Cardiocentro Ticino). A different therapeutical application envisioned the use of EVs as a drug delivery system. A setup for optimal EV loading strategy with chemotherapeutics was presented as a potential treatment for bladder cancer cells, thus increasing delivery efficiency while reducing toxicity (Alessia Brancolini, Università Vita-Salute San Raffaele, Milano, Italy). Not only mammalian cell-derived EVs, but also plant-derived EVs are among the most appealing next-generation biological and industrial agents. EV-like nanoparticles have recently been recognized as bioactive components of ginger (*Zingiber officinale*) and have been proven to hold health-protecting and/or health-enhancing properties in pre-clinical settings, thus prospecting novel tools for delivering effector molecules with encouraging efficacy and safety profiles. Francesca Loria from HansaBiomed Life Sciences (Tallinn, Estonia) showed how to overcome the challenges in isolation and purification of ginger-derived nanoparticles (GDNs). She investigated an enzyme-assisted ginger rhizome cell wall digestion to promote GDN extraction from the apoplast.

The diagnostic potential of circulating EVs was mainly approached by technical reports showing the implementation of a strategy for targeting and analyzing EVs directly embedded in complex biofluids such as serum, plasma, urine, *etc*., thus allowing the exploitation of “ease-of-use” and possible “close to point-of-care” assays for the detection of the disease at the earliest possible stage. In this context, two digital detection platforms were compared side by side: the ExoView® Analyzer, which is based on the principle of single particle interferometric reflectance imaging sensing (SP-IRIS), and Quanterix Simoa® Technology, based on the single-molecule array technology (Simoa). Sensitivity in immune phenotyping of a well-characterized EV sample has been studied, as well as possible applicative implications and rationales for alternative or complementary use of the two platforms in biomarker discovery or validation. With throughput capability and level of automatization, Simoa seems to be the most suitable platform in clinical validation settings (Roberto Frigerio, Consiglio Nazionale Delle Ricerche, Milano, Italy). As an alternative method to detect EV-enclosed miRNA-638 which is associated with the risk of ischemic stroke^[6]^, Ana Rubio-Monterde (Paperdrop Diagnostics, Barcelona, Spain) showed the potential of a new approach consisting of an isothermal amplification followed by a read-out in a rapid diagnostic test (RDT). The isothermal amplification method used needs minimal sample preparation and worked optimally at a temperature of around 37 °C-42 °C. After 35 min of reaction time, the amplified product is applied over a test strip with a buffer, where the test result can be read after 5 min, giving a turnaround time of 40 min. An innovative approach presented during the oral section was using Fourier Transform Infrared Spectroscopy (FTIR) to directly access EV characterization for diagnostic and classification purposes using absorption bands of biomolecules. The authors showed that FTIR in the mid-IR range was used to investigate the composition of EV origin and allowed the discrimination of serum-derived EVs from patients diagnosed with hepatocellular carcinoma from healthy subjects. They showed that EVs could be classified with high accuracy, precision, specificity, and sensitivity using logistic regression and PCA based on characteristic mid-IR bands (Sabrina Romanò, Università Cattolica Del Sacro Cuore, Rome, Italy). Andrea Zendrini from the Università Degli Studi di Brescia (Brescia, Italy) showed the results of the first attempt to quantify the surface-to-bulk partition of proteins in EVs. He established a semi-quantitative model based on microstructural data to estimate the overall protein content of an EV as well as of the partition between membrane (surface) associated and lumen (bulk) contained proteins as a function of the EV size. The model was successfully tested to analyze and describe a real preparation composed of subpopulations of small EVs and large oncosomes from human prostate cancer cells.

Technical reports on EV fractionating included the development of a protocol based on the asymmetrical flow field-flow fractionation (A4F) technique to separate different-sized EV subpopulations from the synovial fluid (SF). The authors showed that they were able to isolate four subpopulations of EVs with a radius ranging from 20 to 700 nm with a differential profile when comparing EVs isolated from the arthritic or inflamed SF and those isolated from the healthy SF (Daniele D’Arrigo, Ente Ospedaliero Cantonale, Bellinzona, Switzerland). A further innovative approach to isolating small EVs is a strategy implementing membrane-sensing proteins as convenient, easy-to-synthesize novel molecular probes for targeting highly curved membranes. Starting from a previously identified class of membrane-sensing peptides derived from Bradykinin protein, an atomic-scale molecular dynamics (MD) simulation was presented to provide an enhanced understanding of the interactions involved in membrane sensing. Using an “atom’s eye view” of the system peptide/membrane, the authors carefully designed a series of simulations, which followed the peptide and the membrane in atomic detail [Alessandro Strada, National Research Council, Istituto “Giulio Natta” (SCITEC-CNR), Milan, Italy].

## POSTER SESSION

Within the poster session, twelve posters were shown and presented mainly by young scientists. It was a huge challenge to select a poster for the EVCNA Prize. The topics of the posters covered the whole area of basic research. They addressed the methodology of EV purification and analysis, as well as the role of EVs as biomarkers and therapeutic agents in patho-physiological processes. The poster voting committee finally reached a decision and selected the poster entitled “Development of breakthrough liquid biopsy diagnostic via novel exosomal biomarkers for patient stratification in prostate cancer” by Alekhya Mazumdar (University Hospital of Zürich, Zürich, Switzerland) as “Best Poster Presentation”.

## CONCLUSION

Overall, the *2nd Lugano ExoDay* provided a fruitful platform for establishing interactions among the participants, especially for the young scientist community, who could take advantage of free registration fees that contributed to the success of the event. Intense discussions accompanied the talk and poster sessions on the molecular and functional characterization of EVs and the refinement of strategies for therapeutic applications. The meeting was an exceptional opportunity for research groups working on EVs in Switzerland to share the progress report of their own research to remain up-to-date with the advances in the field of EVs. Such initiative may pave the way for a Swiss-based task force of researchers and scientists involved in this specific field to establish future collaborative research centers focusing on EV science and the implementation of EV core facilities.

A short film of the day can be seen at (MARVEL project symposium 13 April 2022, Lugano, Switzerland - YouTube: https://www.youtube.com/watch?v=_tkoyZOBOGY)

The *Lugano ExoDay* task force, on behalf of all institutions taking part in this special event, thanks all contributors, the sponsors, and the organizing team for their efforts in making this meeting a success. We are all looking forward to our third edition, taking place in spring 2023 in Lugano.

## DECLARATIONS

### Copyright

© The Author(s) 2022.
